# Post-marketing safety concerns with foscarbidopa/foslevodopa: A pharmacovigilance study with disproportionality analysis based on FAERS

**DOI:** 10.1097/MD.0000000000048874

**Published:** 2026-05-15

**Authors:** Yuchen Peng, Aili Ding, Shihong Zhang, Fanli Kong, Ling Wu, Weijia Sun, Zhengwu Sun, Xinkuo Zheng, Yalin Xi

**Affiliations:** a Department of Pharmacy, Central Hospital of Dalian University of Technology, Dalian, China.

**Keywords:** adverse events, drug safety, ParkinsonFAERS, Foscarbidopa/foslevodopa, Parkinson disease, signal detection

## Abstract

Foscarbidopa/foslevodopa, a 24-hour subcutaneous infusion of levodopa/carbidopa prodrugs, is approved for advanced Parkinson disease with motor fluctuations. Due to limited post-marketing data, the association between this novel formulation and related adverse events (AEs), particularly infusion-related or neurological complications, remains underexplored. This study aims to identify potential safety signals of foscarbidopa/foslevodopa-associated AEs using data mining of the FDA Adverse Event Reporting System (FAERS). To quantify safety risk signals of foscarbidopa/foslevodopa, disproportionality analyzes were conducted using the reporting odds ratio and Bayesian Confidence Propagation Neural Network methods, based on FAERS data from the last quarter of 2024 (2024Q4) to the second quarter of 2025 (2025Q2). Signal thresholds were strictly defined, and sensitivity analyses were performed to verify the robustness of results; subgroup analyses were conducted to explore sex differences and severity-related characteristics of AEs. The FAERS recorded 7527 foscarbidopa/foslevodopa-related AEs affecting 1914 patients. A total of 1616 patients experienced serious AEs, including 265 deaths. The most frequently reported AEs were on and off phenomenon (n = 223), fall (n = 195), hallucination (n = 156), and dyskinesia (n = 132). Potential safety signals were detected, such as on and off phenomenon, hallucination, and infusion site infection. Notably, FAERS data are subject to spontaneous reporting bias, missing information, and lack of denominator exposure data, so the above results only reflect statistical associations rather than definite causal relationships. FAERS data identify potential clinically significant associative AEs with foscarbidopa/foslevodopa, such as infusion site reactions and infections and hallucination. These findings support the need for enhanced monitoring of high-risk patients (e.g., mental and behavioral disorders, skin disorders) and reinforce the importance of real-world surveillance for novel Parkinson disease therapies. All findings are hypothesis-generating and require further clinical validation to confirm causal relationships.

## 1. Introduction

Foscarbidopa/foslevodopa is a 24-hour continuous subcutaneous infusion formulation composed of phosphate ester prodrugs of carbidopa and levodopa (trade name: Vyalev).^[1]^ Approved by the U.S. Food and Drug Administration (FDA) in October 2024 for the treatment of motor fluctuations in adults with advanced Parkinson disease (PD), it represents the first levodopa-based therapy to enable continuous subcutaneous delivery.^[2,3]^ As prodrugs, foscarbidopa and foslevodopa are cleaved by tissue phosphatases after subcutaneous administration to release active carbidopa and levodopa; carbidopa inhibits peripheral levodopa decarboxylation to increase central levodopa bioavailability, and continuous infusion maintains stable plasma levodopa concentrations to alleviate motor fluctuations.^[1]^Its approval was supported by results from the pivotal phase III clinical trial (NCT04380142), which enrolled 141 patients and demonstrated a significant extension of daily “on” time without troublesome dyskinesia (2.72 hours vs oral therapy 0.97 hours), with substantially improved bioavailability compared to conventional oral formulations.^[4]^

Safety data supporting its approval were primarily derived from clinical trials, where the majority of AEs are mild to moderate.^[5]^ A phase III clinical trial showed that the most frequent AEs are infusion site AEs (erythema 27%, pain 26%, cellulitis 19% and edema 12%). The only system organ class that had more than one serious adverse event was infections and infestations (catheter site cellulitis 1% and infusion site cellulitis 1%).^[4]^

Spontaneous reporting systems, such as the FDA Adverse Event Reporting System (FAERS), provide a critical and accessible resource for identifying safety concerns in real-world clinical settings post-marketing. While inherent limitations include reporting bias, data quality issues, and the inability to calculate incidence rates, post-marketing pharmacovigilance remains pivotal for monitoring medication safety and detecting novel rare signals. To date, discussions on the safety of foscarbidopa/foslevodopa have been limited to clinical trial results, and no systematic real-world pharmacovigilance studies have been conducted to characterize its adverse event profile, detect potential safety signals, and analyze the clinical characteristics of associated AEs since its FDA approval.^[1,6,7]^ This study aimed to systematically detect potential safety signals of foscarbidopa/foslevodopa-associated AEs using disproportionality analysis of FAERS data, and further characterize the clinical features (e.g., severity, sex differences, time-to-onset) of these events, thereby providing evidence to support its safe clinical use.

## 2. Method

### 2.1. Data source and data processing

**Figure 1.**
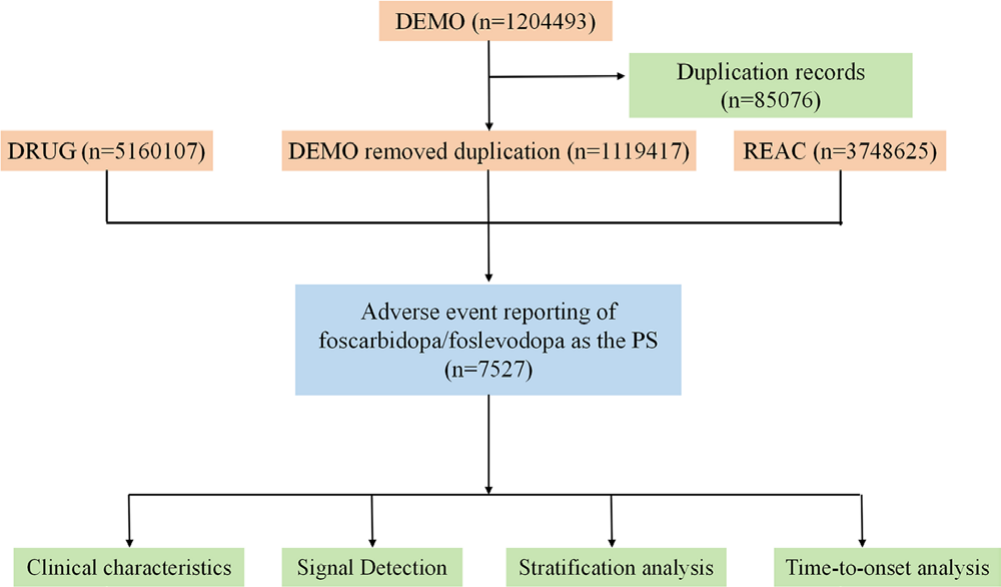
Flow diagram of this study. DEMO = demographic information; DRUG drug, information; REAC, adverse events; PS = primary suspect drug.

The FAERS database comprises 7 core datasets: patient demographics (DEMO), drug/biologic information (DRUG), adverse events (REAC), patient outcomes (OUTC), report sources (RPSR), start/end dates of drug therapy (THER), and indications for drug (INDI).^[8]^ These files are interconnected via unique identifiers (primaryid as the primary unique identifier, caseid as the secondary association identifier) to enable relational data analysis. Notably, reports removed by regulatory authorities or pharmaceutical sponsors are stored separately in Deleted files and were excluded upfront. The rule for data cleaning is to remove duplicate reports by following the method recommended by the FDA for data de-duplication: reports with the same primaryid, patient gender, age, drug exposure time, and AE description were defined as duplicate reports and only the first record was retained.^[9]^ A schematic overview of the data extraction, cleaning, and analytical workflow is presented in Figure 1.

We searched the reports submitted to FAERS between October 1, 2024 and June 30, 2025. To identify foscarbidopa/foslevodopa-associated reports, the DRUG dataset was queried using generic identifiers (foscarbidopa\foslevodopa in drugname and prod_ai fields) and the trade name Vyalev (in drugname). Only reports where the medication was designated as the primary suspect (PS) (role_cod = “PS”) were included to strengthen the statistical association between the drug and reported adverse events. Adverse events were coded using preferred terms (PT) from the Medical Dictionary for Regulatory Activities (MedDRA v27.0).^[10]^ Given the drug’s subcutaneous delivery and central nervous system activity, this analysis focused on PT within the SOC “General disorders and administration site conditions” and “Nervous system disorders”; other SOCs were screened as supplementary references to avoid missing potential safety signals.

### 2.2. Statistical analysis

Since no single standardized method is universally accepted for pharmacovigilance signal detection, this study utilized 2 disproportionality analysis techniques: the reporting odds ratio (ROR) and the Bayesian Information Component (IC), each with 95% confidence intervals (CI) computed to assess significance.^[11–13]^ A signal was deemed statistically significant for ROR when the lower limit of the 95% CI exceeded 1 with a minimum of 3 reports, and for IC when the lower limit of the 95% CI was >0. The ROR method was prioritized for its simplicity and common application in spontaneous reporting databases, while the Bayesian IC approach was included to address potential confounding and improve detection of stronger associations.^[14]^ To ensure reliability, an adverse event was classified as a confirmed safety signal only if both analytical methods independently satisfied their significance criteria. It should be explicitly stated that disproportionality analysis is a statistical method to identify abnormal reporting frequency of AEs, which cannot control for confounding factors (e.g., disease severity, concomitant drugs) and cannot prove a definite causal relationship between drugs and AEs. In addition, due to the large number of PTs screened in this study, there is a potential risk of false positive signals caused by multiple testing; thus, only PTs with both ROR and IC meeting the signal criteria were defined as potential safety signals to reduce false positives. The 2 algorithm formulas and threshold standards mentioned above are shown in Table 1.

**Table 1:** Calculation formula for detecting AE signals related to foscarbidopa/foslevodopa and positive signal determination criteria.

Algorithm	Formula	Positive signal determination threshold
ROR	ROR = ad/bc $95\;\%\;\;\;CI=e^{ln\left(ROR\right)\pm 1.96\sqrt{1/a+1/b+1/c+1/d}}$	n ≥ 3, 95%CI>1
BCPNN	χ^2^ = (ad-bc)^2^(a + b + c + d)/ [(a + b)(c + d)(a + c)(b + d)] IC = log_2_[a (a + b + c + d)/ ((a + c)(a + b))] $IC025=e^{ln\left(IC\right)-1.96\sqrt{1/a+1/b+1/c+1/d}}$	IC025>0

AE = adverse event, CI = confidence interval, ROR = reporting odds ratio.

### 2.3. Secondary analysis

We also observed disproportionate reporting for females and males separately. We compared age, sex, time-to-onset of AEs, and concomitant use of other anti-Parkinson drugs between patients with serious and non-serious AE reports. We additionally evaluated the association between serious AEs and the concomitant use of aspirin, acid-suppressing drugs, statins, antidepressants, or benzodiazepines, these drug classes were selected for the following clinical rationales: they are the most commonly co-prescribed drugs in PD patients in clinical practice (aspirin for cardiovascular prophylaxis, acid-suppressing drugs for levodopa-induced gastrointestinal irritation, statins for dyslipidemia, and antidepressants/benzodiazepines for neuropsychiatric comorbidities); existing studies have shown that some of these drugs may interact with levodopa metabolism (e.g., acid-suppressing drugs alter gastric pH and affect levodopa absorption; antidepressants may exacerbate dopaminergic-related neuropsychiatric AEs).^[15,16]^ Chi-square (χ2) test was used to analyze the proportions, while the age comparison adopted the Mann–Whitney U test (M-W-U). A “volcano plot” was created to visualize the differences in the severity of AEs and gender aspects.

## 3. Results

### 3.1. Descriptive analysis

A total of 7527 AEs related to foscarbidopa/foslevodopa were identified, involving 1914 patients with an average of 3.9 AEs per patient. Among these patients, 1616 experienced serious AEs, including 265 deaths. It should be noted that FAERS is a spontaneous reporting system with reporting bias (serious AEs are more likely to be reported) and lack of denominator exposure data, so the proportion of serious AEs cannot reflect the actual incidence in clinical practice. Age data were available for 1475 patients, with a mean age of 70.6 ± 9.5 years, and 1046 patients were male (Table 2). The characterization of deaths is provided in Table 3.

**Table 2:** Characteristics of the patients submitted to the US FDA Adverse Event Reporting System for foscarbidopa/foslevodopa.

Characteristics	No (%)
Gender	
Female	751 (39.24)
Male	1046 (54.65)
Not specified	117 (6.11)
Age, y	
18–44	16 (0.84)
45–64	334 (17.45)
65–74	574 (29.99)
≥75	551 (28.79)
Not specified	439 (22.94)
Reporting year	
2024	239 (12.49)
2025	1675 (87.51)
Reporter	
Consumer	1073 (56.06)
Physician	348 (18.18)
Pharmacist	25 (1.31)
Other health professional	466 (24.35)
Not specified	2(0.10)
Reporting area	
Europe	1299 (67.87)
North America	453 (23.67)
Asia	160 (8.36)
Oceania	2 (0.10)
Patients with serious and non-serious reports	
Non-serious	298 (15.57)
Serious	1616 (84.43)
Reported outcome*	
Death	265 (13.85)
Nonfatal: hospitalization, disability, or life-threatening	1090 (56.95)
Nonfatal: other serious outcome	531 (27.74)
Time-to-onset, d†	
0–30 d	218 (11.39)
31–60 d	82 (4.28)
61–90 d	55 (2.87)
91–120 d	25 (1.31)
121–150 d	22 (1.15)
151–180 d	14 (0.73)
181–360 d	11 (0.57)

AE = adverse event.

* The patients can have multiple serious AE outcomes.

† The time from the start of therapy to the date the event occurred.

**Table 3:** Characterisation of deaths for US Adverse Event Reports submitted to the US FDA Adverse Event Reporting System for foscarbidopa/foslevodopa.

Characteristics	No (%)
Total death cases	265 (100.00)
Gender	
Female	84 (31.70)
Male	161 (60.75)
Not specified	20 (7.55)
Age, y	
≤ 64	24 (9.06)
65–74	67 (25.28)
≥ 75	126 (47.55)
Not specified	48 (18.11)
Reporter	
Consumer	148 (55.85)
Physician	37 (13.96)
Pharmacist	3 (1.13)
Other health professional	77 (29.06)
Top 10 causes of death according to SOC	
General disorders and administration site conditions	112 (19.61)
Nervous system disorders	96 (16.81)
Infections and infestations	67 (11.73)
Psychiatric disorders	39 (6.83)
Respiratory, thoracic and mediastinal disorders	38 (6.65)
Injury, poisoning and procedural complications	36 (6.3)
Cardiac disorders	34 (5.95)
Gastrointestinal disorders	29 (5.08)
Metabolism and nutrition disorders	20 (3.5)
Surgical and medical procedures	18 (3.15)
Top 10 causes of death according to PT	
General physical health deterioration	29 (5.08)
Pneumonia	22 (3.85)
Parkinson disease	19 (3.33)
Cardiac arrest	14 (2.45)
Fall	13 (2.28)
Hallucination	11 (1.93)
Pneumonia aspiration	10 (1.75)
Pyrexia	9 (1.58)
Urinary tract infection	8 (1.4)
On and off phenomenon	8 (1.4)
Time-to-onset, d^*^	
0–30 d	17 (28.81)
31–60 d	14 (23.73)
61–90 d	16 (27.12)
91–120 d	5 (8.47)
121–150 d	4 (6.78)
151–180 d	2 (3.39)
181–360 d	1 (1.69)

PT = preferred term, SOC = system organ class.

* The time from the start of therapy to the date of death.

**Figure 2.**
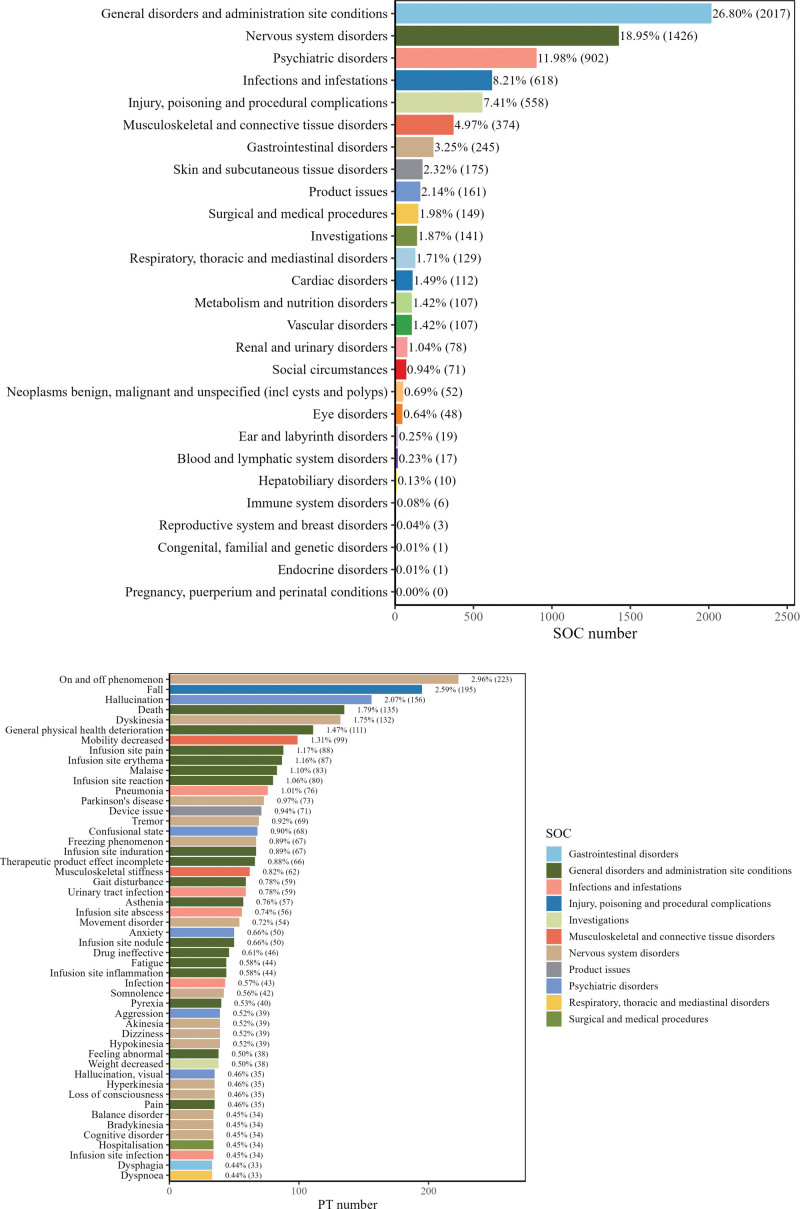
The number of reports corresponding to the SOC and the top 50 PT for adverse reaction events related to foscarbidopa/foslevodopa. SOC = system organ class; PT = preferred term.

After screening 1914 reports, the frequencies and proportions of SOC and the top 50 PTs are shown in Figure 2. Detailed data on the frequency and onset time of AEs are presented in Table S1, which lists the top 60 most common AEs and their mean time-to-onset.

### 3.2. Disproportionality analysis

Table S2 shows disproportionate estimates of the top 60 adverse events associated with foscarbidopa/foslevodopa grouped according to 3 predefined categories. The criteria for category classification are as follows: Expected with detected signals: AEs that are mentioned in the foscarbidopa/foslevodopa official label or have been clearly reported in phase III clinical trials, such as on and off phenomenon, hallucination, dyskinesia, and infusion site reaction; disease-expected: AEs that are highly likely to be associated with the natural progression of PD itself rather than drug exposure, such as Tremor, Freezing phenomenon and Gait disturbance; without signal–related: AEs with no statistical signal detected by both ROR and IC methods.

**Figure 3.**
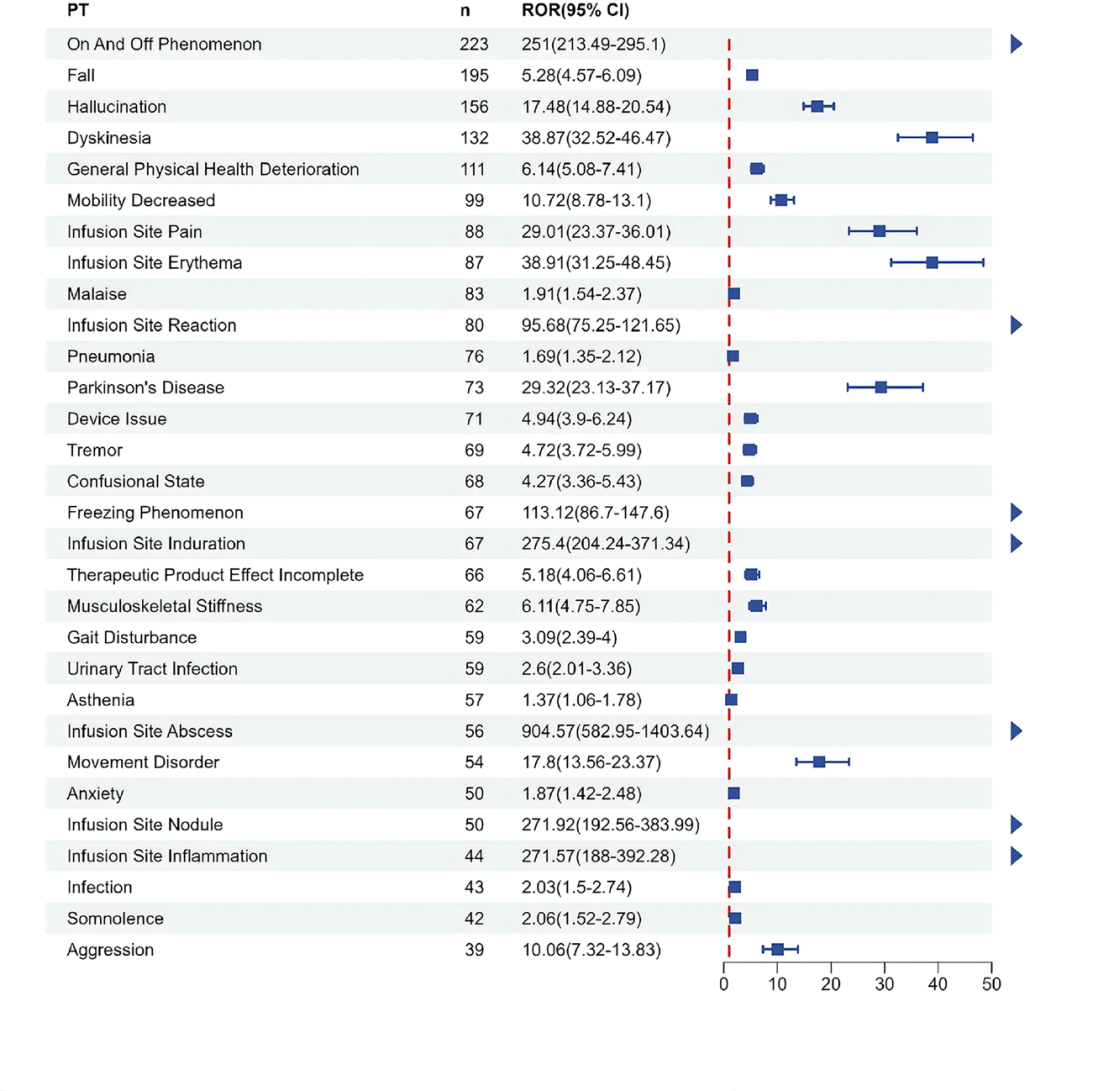
The forest map showing the top 30 positive safety signals using the ROR approach. CI = confidence interval; PT = preferred term; ROR = reporting odds ratio.

Figure 3 is a forest map showing the top 30 reported AEs, which were statistically significant. Potential safety signals were detected for a large number of AEs, such as on and off phenomenon, fall, hallucination, dyskinesia, infusion site pain. In addition, among all foscarbidopa/foslevodopa related AEs with positive signals counted more than 5 times, potential unexpected AE signals included dehydration, pulmonary embolism, epilepsy, volvulus, and multiple fracture types (femoral neck, hip, femur, upper limb, spinal).

### 3.3. Sex differences in foscarbidopa/foslevodopa-related AEs

**Figure 4.**
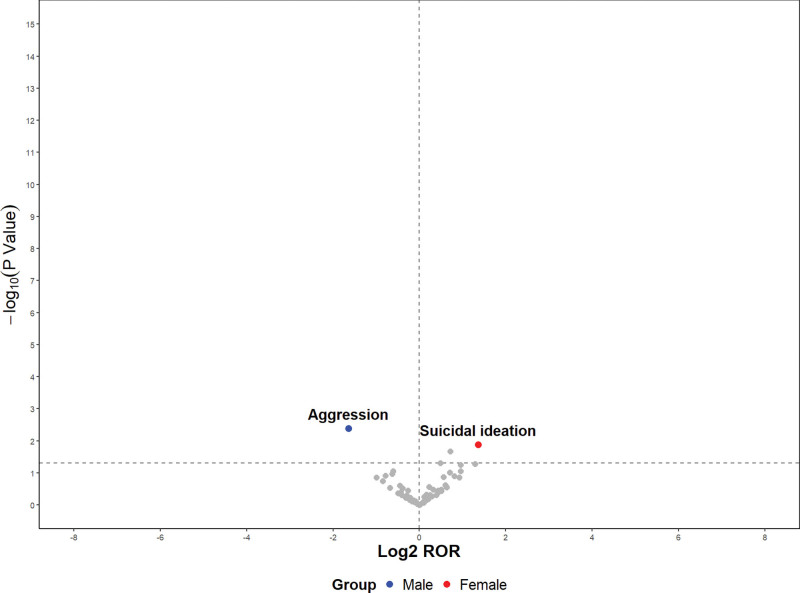
The volcano plot to visualize the differences in sex. ROR = reporting odds ratio; the horizontal axis is Log2 ROR, where values > 0 indicate that the AE is more likely to be reported in the corresponding gender; values < 0 indicate the opposite. The vertical axis is -log10 (*P* Value), reflecting the statistical significance of the difference.

The most common safety signals were similar in males and females. However, Aggression was more likely to be reported in male patients, whereas Suicidal ideation was more likely to be reported in female patients (*P* < .05) (Fig. 4; Table S3a, b).

### 3.4. Serious versus non-serious AEs

Compared with patients without serious AEs, those with serious AEs were more likely to receive multiple anti-Parkinson drugs (*P* < .001) and had a significantly different age distribution (*P* < .001): the non-serious AE group had a higher proportion of patients aged 45 to 64 years (*P* < .001), while the serious AE group had more patients aged ≥ 75 years (*P* < .001). No significant differences were observed between the 2 groups in the concomitant use of aspirin, acid-suppressing medications, statins, antidepressants, or benzodiazepines, nor in sex distribution (*P* = .268).

**Figure 5.**
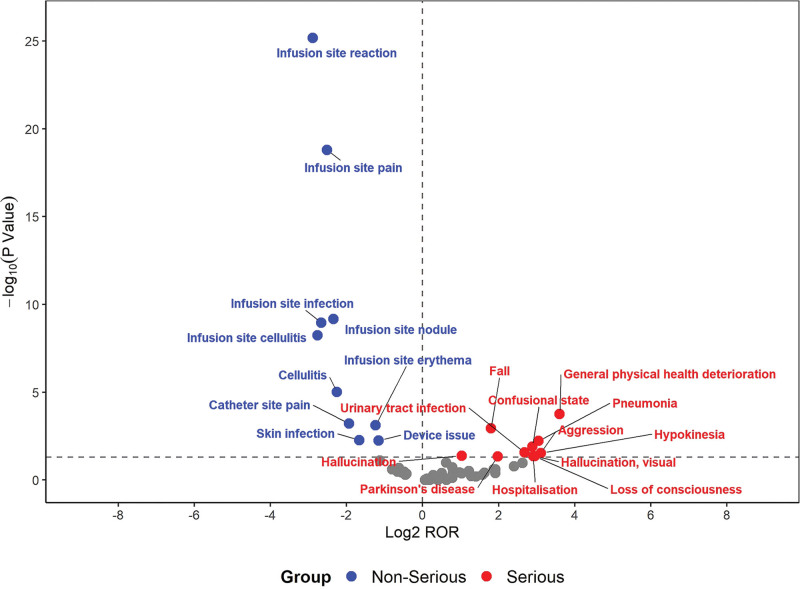
The volcano plot to visualize the differences in AE severity. AE = adverse event, ROR = reporting odds ratio; the horizontal axis is Log_2_ ROR, where values > 0 indicate that the AE is more likely to be reported as serious; values < 0 indicate the AE is more likely to be reported as non-serious. The vertical axis is -log_10_ (*P* Value), reflecting the statistical significance of the difference.

Fall, hallucination, general physical health deterioration, pneumonia, Parkinson disease, confusional state, urinary tract infection, aggression, hypokinesia, hallucination, visual, loss of consciousness, hospitalization were more likely to be reported as serious. In contrast, infusion site pain, infusion site erythema, infusion site reaction, device issue, infusion site nodule, infusion site infection, cellulitis, infusion site cellulitis, catheter site pain, skin infection were more likely to be reported as non-serious (Fig. 5).

### 3.5. Time-to-onset analysis

**Figure 6.**
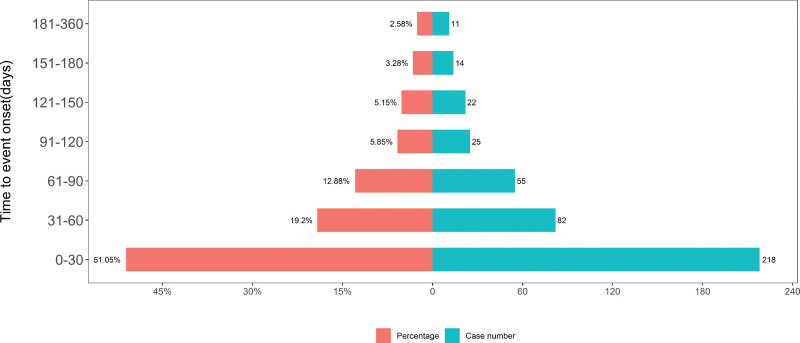
Cumulative incidence of foscarbidopa/foslevodopa-related adverse events.

**Figure 7.**
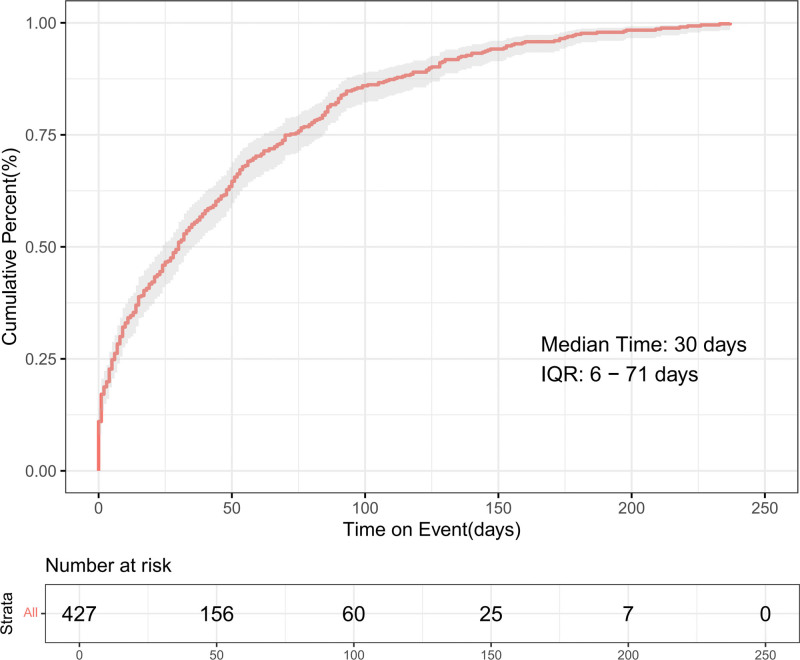
Time to onset distribution of foscarbidopa/foslevodopa-related adverse events. IQR = interquartile range.

The onset times of foscarbidopa/foslevodopa-associated AEs were collected from the database. Excluding unreported or incorrect reports, a total of 1210 AEs reported onset time. The median onset time was 30 days (interquartile range [IQR] 6–71 days). As shown in Figures 6–7, most AEs occurred within the first 3 months after initiation: 51.05% within the first month, 19.20% in the second month, and 12.88% in the third month. It should be noted that the time-to-onset data may be affected by incomplete or delayed reporting in the FAERS database, and the results only reflect the reported situation rather than the actual clinical onset characteristics.

### 3.6. Sensitivity analysis

We observed that some data in the FAERS system are missing or not available. For example, age is missing in 22.94% of reports and clinical outcome is missing in 15.57% of reports. To reduce the potential impact of these missing values on the study results to the greatest extent possible, sensitivity analyses were conducted. For age and clinical outcome, we excluded records that contained missing data and then implemented sensitivity analysis for each variable. Then, we further excluded the influence of combined medication and only retained the data of those using foscarbidopa/foslevodopa for conducting the sensitivity analysis. Table 4 presents the top 10 positive PT signals from the sensitivity analysis. Upon comparison, while the ranking of positive PT signal strength changed, no fundamentally different outcomes were observed, indicating that the study results have good robustness.

**Table 4:** Sensitivity analysis of positive PT signals.

PT	No.	ROR	Lower 95%CI	Upper 95%CI	IC	Lower 95%CI	Upper 95%CI
Exclude missing age							
On and off phenomenon	206	260.56	218.02	311.40	7.24	7.09	7.39
Fall	180	5.35	4.61	6.21	2.37	2.16	2.58
Hallucination	139	23.56	19.82	28.01	4.45	4.22	4.68
Dyskinesia	123	39.91	33.09	48.13	5.15	4.91	5.39
General physical health deterioration	98	7.96	6.51	9.74	2.95	2.67	3.23
Mobility decreased	94	11.76	9.56	14.45	3.50	3.21	3.78
Malaise	78	2.08	1.66	2.60	1.04	0.73	1.36
Infusion site erythema	67	33.27	25.89	42.76	4.92	4.60	5.25
Infusion site pain	66	26.50	20.62	34.05	4.62	4.29	4.96
Parkinson disease	66	36.82	28.56	47.46	5.06	4.73	5.39
Exclude missing outcome							
On and off phenomenon	208	173.23	146.88	204.31	6.87	6.71	7.03
Fall	189	3.90	3.37	4.51	1.92	1.72	2.13
Hallucination	148	12.65	10.72	14.93	3.59	3.36	3.82
Dyskinesia	121	27.04	22.45	32.56	4.64	4.39	4.88
General physical health deterioration	110	4.65	3.84	5.61	2.18	1.92	2.45
Mobility decreased	93	7.68	6.25	9.45	2.90	2.61	3.19
Malaise	77	1.35	1.08	1.69	0.43	0.11	0.75
Pneumonia	75	1.27	1.01	1.59	0.34	0.02	0.66
Parkinson disease	71	21.76	17.11	27.67	4.35	4.03	4.68
Infusion site erythema	69	23.16	18.14	29.56	4.44	4.11	4.77
Excluded combined medication							
Fall	87	6.85	5.52	8.49	2.72	2.42	3.01
Hallucination	51	12.89	9.73	17.08	3.62	3.24	4.01
On and off phenomenon	50	634.60	409.79	982.74	7.98	7.73	8.23
Infusion site reaction	48	574.93	372.57	887.20	7.92	7.66	8.18
General physical health deterioration	47	5.72	4.28	7.65	2.48	2.07	2.89
Infusion site pain	40	122.82	86.25	174.89	6.56	6.17	6.95
Infusion site erythema	39	136.25	94.85	195.72	6.68	6.28	7.07
Dyskinesia	38	48.28	34.45	67.67	5.42	4.99	5.85
Pneumonia	35	2.35	1.68	3.28	1.22	0.74	1.69
Device issue	31	3.66	2.57	5.23	1.85	1.35	2.35

CI = confidence interval, IC = information component, PT = preferred term, ROR = reporting odds ratio.

## 4. Discussion

This pharmacovigilance study comprehensively and systematically presented the most recent findings regarding the potential safety profile of foscarbidopa/foslevodopa based on post-marketing data from the FAERS database. Since its approval, the number of reported cases has increased sharply on a quarterly basis. In the first quarter following approval (2024Q4), FAERS recorded 239 reports (approximately 79.7 per month). By 2025Q1, this number had risen to 570 reports (approximately 190.0 per month), and by 2025Q2, it reached 1105 reports (approximately 368.3 per month). If the Weber effect continues to apply, peaking approximately 2 years after approval, the number of reports related to foscarbidopa/foslevodopa is expected to rise further, indicating potential emerging safety concerns.^[17,18]^ This upward trend may be partially attributed to increased drug utilization following its market launch.^[1,19]^ Moreover, the drug has garnered significant attention within both the scientific community and the general public, which may heighten safety awareness among healthcare professionals and patients, potentially contributing to reporting bias. These observations underscore the importance of ongoing epidemiological surveillance and post-marketing safety evaluations.

Our analysis of 7527 foscarbidopa/foslevodopa-related AEs reveals a substantial burden of AEs, with an average of 3.9 events per patient among 1914 individuals. The high proportion of serious AEs (1616 cases, including 265 fatalities) highlights the need for careful monitoring of patients receiving this therapy in clinical practice, particularly regarding infusion site events which are commonly reported with continuous subcutaneous administration.^[20,21]^ The demographic profile of affected patients, with a mean age of 70.6 years and male predominance (1046 patients), reflects the typical Parkinson disease population,^[22]^ suggesting that the observed AE pattern may represent the drug’s real-world associative safety profile in its intended patient population.

The spectrum of most frequently reported AEs aligns with both the pharmacological action of levodopa and the expected complications of continuous subcutaneous infusion therapy.^[4,23]^ The on-off phenomenon (2.96%), falls (2.59%), and hallucinations (2.07%) represent well-established complications of dopaminergic therapy, while infusion site reactions (e.g., pain, erythema, induration) reflect the expected local complications associated with continuous subcutaneous drug delivery.^[24]^ The variable time-to-onset patterns, ranging from early infusion site reactions (mean 8.93 days) to later motor complications (mean 59.41 days for on-off phenomenon), provide valuable insights for clinical monitoring schedules and patient education.

More importantly, this analysis identified several potential unexpected AE signals that were not reported in regulatory trials, including dehydration, pulmonary embolism, various fractures (femoral neck, hip, femur, upper limb, spinal), epilepsy, and volvulus, thereby raising important safety concerns. These findings may suggest previously unrecognized potential risks associated with foscarbidopa/foslevodopa therapy. However, these associations should be interpreted with caution due to the inherent limitations of FAERS data, including underreporting, missing data, and confounding factors. The absence of denominator data precludes incidence estimation, and the observed signals may reflect disease-related factors rather than drug effects. Parkinson disease has been reported to be associated with an increased risk of fractures. Therefore, the fracture signals may be related to the decreased bone density in Parkinson patients.^[25,26]^ The fracture signals might also be explained as by the following hypothetical mechanisms: After taking medication, if the motor ability of Parkinson patients improves, the increase in their activity level may lead to falls and fractures; and dehydration may be related to the aggravation of autonomic nerve dysfunction.^[27]^ However, the mechanisms underlying potential associations with pulmonary embolism and volvulus require further investigation. Furthermore, the possible reasons for this could also be attributed to differences between the clinical trial population and the real-world population, small sample size, short follow-up duration, and reporting bias. Therefore, continuous post-marketing surveillance and real-world studies are crucial for identifying these delayed or rare adverse events.

The overall similarity in safety signals between males and females suggests that foscarbidopa/foslevodopa’s safety profile is generally consistent across sexes. However, the identified sex-specific differences in aggression (more common in males) and suicidal ideation (more common in females) provide valuable insights for personalized patient management. These findings may reflect biological differences in drug metabolism (e.g., females have lower clearance and higher bioavailability of levodopa) or neuropsychiatric vulnerability between sexes, or alternatively, they could be influenced by reporting biases or differences in symptom expression. The increased reporting of aggression in males aligns with known patterns of impulse control disorders in Parkinson disease, which typically show male predominance. Conversely, the higher reporting of suicidal ideation in females may reflect either biological vulnerabilities or psychosocial factors.^[28,29]^ These sex-specific patterns emphasize the need for tailored monitoring and management strategies based on patient sex, particularly regarding neuropsychiatric adverse events.

Furthermore, comparing serious adverse events with non-serious adverse events helps to identify high-risk patient groups. Stratified analyzes further highlight vulnerable subgroups. Older patients with Parkinson disease who are treated with multiple anti-Parkinson drugs have a significantly increased risk of experiencing AEs related to foscarbidopa/foslevodopa.^[22,30]^ The significant association between concomitant use of multiple anti-Parkinson drugs and serious AEs suggests that disease severity and complexity, rather than foscarbidopa/foslevodopa itself, may be important determinants of serious adverse outcomes. This finding underscores the challenge of managing advanced Parkinson disease, where multiple medications are often necessary but may contribute to increased AE risk. The age distribution differences between serious and non-serious AE groups provide important insights for risk stratification. The higher proportion of younger patients (45–64 years) in the non-serious group may reflect better physiological reserve and resilience to adverse events, while the predominance of older patients (≥75 years) in the serious AE group highlights the vulnerability of this population due to age-related pharmacokinetic changes, comorbidities, and reduced functional reserve. The differential pattern of AE seriousness provides practical guidance for clinical management. The association of typical Parkinson-related symptoms (falls, hallucinations, confusion) and systemic complications (pneumonia, urinary tract infections) with serious outcomes emphasizes the need for vigilant monitoring of these events. Conversely, the predominance of infusion site-related events in the non-serious category suggests that while these are common, they are generally manageable without serious consequences.

Most AEs related to foscarbidopa/foslevodopa occurred within the first 3 months of treatment (median 30 days, IQR 6–71 days). This result has important clinical implications for monitoring strategies and patient education. The early onset of many AEs (51.05% within first month) suggests that close monitoring is particularly crucial during the initial treatment period. This early phase may represent either acute reactions to the drug or the unmasking of underlying vulnerabilities in Parkinson patients. The subsequent decline in AE incidence over time may indicate either adaptation to therapy, dose adjustments, or discontinuation in patients who experienced significant AEs. The variable timing of different AE types provides insights into potential underlying mechanisms. Early-onset infusion site reactions likely represent local tissue irritation, while later-onset motor complications (such as on-off phenomena) may reflect adaptive changes in the dopaminergic system.^[4,23]^ This temporal pattern can help clinicians differentiate between different types of adverse events and implement appropriate timing-specific monitoring strategies.

An important strength of our study is that it is the first to utilize real data from FAERS to describe the post-marketing safety of foscarbidopa/foslevodopa. This study encompasses a large cohort (1914 patients) and a short post-approval window (9 months) to capture early safety signals. However, this study has several inherent limitations that must be emphasized. As a spontaneous reporting system, FAERS is susceptible to underreporting, reporting bias and potential stimulated reporting bias, with serious adverse events far more likely to be reported than mild ones. This study only identifies statistical associations rather than definite causal relationships between the drug and adverse events, and the lack of denominator exposure data precludes the calculation of actual AE incidence. Additionally, the database has unavoidable data quality issues such as missing age and clinical outcome information; confounding by indication also cannot be excluded, as PD severity and polypharmacy may be major contributors to serious adverse events. Despite strict signal detection criteria, multiple testing may still result in potential false positive safety signals. Despite these challenges, pharmacovigilance research remains crucial for monitoring medication safety and uncovering rare, clinically relevant safety signals that may be underrepresented in pre-approval clinical trials.

## 5. Conclusion

Post-marketing surveillance of foscarbidopa/foslevodopa using FAERS has identified potential clinically relevant AE signals, particularly infusion site complications, hallucinations, falls, and rare thrombo-embolic or fracture events, that were underrepresented in pre-approval clinical trials. Our findings highlight important potential risk stratification factors, such as advanced age, polypharmacy, and specific demographic characteristics, which may influence both the occurrence and severity of adverse events. The early onset of most adverse events, particularly within the first 3 months of treatment initiation, underscores the necessity for close monitoring during the initial phase of therapy. These results offer valuable hypothesis-generating guidance for clinicians in terms of patient selection, monitoring protocols, and management strategies aimed at enhancing the safety profile of foscarbidopa/foslevodopa in real-world clinical practice. Furthermore, the findings support the adoption of risk-minimization strategies, including pretreatment assessments of skin integrity and cognitive function, structured follow-up during the first 90 days of therapy, and comprehensive patient education regarding early warning signs.^[31]^ Ongoing real-world data collection and further clinical studies are crucial for refining the benefit-risk profile, validating the causal relationships of the identified potential safety signals, and ensuring the safe incorporation of this first-in-class continuous levodopa therapy into standard Parkinson disease treatment management.

## Acknowledgments

We are deeply grateful to the Central Hospital of Dalian University of Technology for their substantial support throughout this study. Additionally, we would like to extend our gratitude to all the editors and reviewers who have contributed their efforts to this research.

## Author contributions

**Data curation:** Yuchen Peng.

**Investigation:** Yuchen Peng.

**Supervision:** Yalin Xi.

**Writing – original draft:** Yuchen Peng.

**Writing – review & editing:** Yuchen Peng, Aili Ding, Shihong Zhang, Fanli Kong, Ling Wu, Weijia Sun, Zhengwu Sun, Xinkuo Zheng, Yalin Xi.








