# Supplementary material for: Post-marketing safety concerns with foscarbidopa/foslevodopa: A pharmacovigilance study with disproportionality analysis based on FAERS
Source: Medicine (Baltimore). 2026 May 15;105(20):e48874. doi: 10.1097/MD.0000000000048874 (PMC13183012; doi:10.1097/MD.0000000000048874)
Supplement: Supplementary file 2 [file medi-105-e48874-s002.docx]

Table S2 Top 60 PT safety signals for adverse reaction events related to foscarbidopa/foslevodopa.

| Groups | PT | No. | ROR | Lower  95%CI | Upper 95%CI | IC | Lower  95%CI | Upper 95%CI | Time-to onset |
| --- | --- | --- | --- | --- | --- | --- | --- | --- | --- |
| Expected with signal* | On and off phenomenon* | 223 | 251 | 213.49 | 295.1 | 7.36 | 7.2 | 7.51 | 59.41 |
|  | Fall* | 195 | 5.28 | 4.57 | 6.09 | 2.36 | 2.16 | 2.56 | 52.81 |
|  | Hallucination* | 156 | 17.48 | 14.88 | 20.54 | 4.05 | 3.83 | 4.27 | 41.06 |
|  | Dyskinesia* | 132 | 38.87 | 32.52 | 46.47 | 5.15 | 4.92 | 5.39 | 28.57 |
|  | Infusion site pain* | 88 | 29.01 | 23.37 | 36.01 | 4.76 | 4.47 | 5.06 | 8.93 |
|  | Infusion site erythema* | 87 | 38.91 | 31.25 | 48.45 | 5.16 | 4.87 | 5.45 | 41.2 |
|  | Malaise* | 83 | 1.91 | 1.54 | 2.37 | 0.92 | 0.62 | 1.23 | 43 |
|  | Infusion site reaction* | 80 | 95.68 | 75.25 | 121.65 | 6.32 | 6.03 | 6.6 | 18.31 |
|  | Pneumonia* | 76 | 1.69 | 1.35 | 2.12 | 0.75 | 0.42 | 1.07 | 59.42 |
|  | Device issue* | 71 | 4.94 | 3.9 | 6.24 | 2.28 | 1.95 | 2.61 | 39.88 |
|  | Confusional state* | 68 | 4.27 | 3.36 | 5.43 | 2.07 | 1.73 | 2.41 | 19.5 |
|  | Infusion site induration* | 67 | 275.4 | 204.24 | 371.34 | 7.46 | 7.19 | 7.74 | 89.5 |
|  | Therapeutic product effect incomplete* | 66 | 5.18 | 4.06 | 6.61 | 2.35 | 2.01 | 2.69 | 54.71 |
|  | Urinary tract infection* | 59 | 2.6 | 2.01 | 3.36 | 1.37 | 1 | 1.73 | 35.45 |
|  | Infusion site abscess* | 56 | 904.57 | 582.95 | 1403.64 | 8.32 | 8.1 | 8.55 | 79.75 |
|  | Anxiety* | 50 | 1.87 | 1.42 | 2.48 | 0.9 | 0.5 | 1.3 | 45.67 |
|  | Infusion site nodule* | 50 | 271.92 | 192.56 | 383.99 | 7.45 | 7.13 | 7.77 | 90.2 |
|  | Infusion site inflammation* | 44 | 271.57 | 188 | 392.28 | 7.45 | 7.11 | 7.8 | 81.5 |
|  | Infection* | 43 | 2.03 | 1.5 | 2.74 | 1.01 | 0.58 | 1.44 | 15.33 |
|  | Somnolence* | 42 | 2.06 | 1.52 | 2.79 | 1.03 | 0.6 | 1.47 | 49.25 |
|  | Aggression* | 39 | 10.06 | 7.32 | 13.83 | 3.3 | 2.85 | 3.75 | 59.59 |
|  | Akinesia* | 39 | 253.05 | 172.03 | 372.21 | 7.39 | 7.02 | 7.75 | 31.55 |
|  | Feeling abnormal* | 38 | 1.98 | 1.44 | 2.72 | 0.98 | 0.52 | 1.43 | 30.2 |
|  | Hallucination, visual* | 35 | 13.9 | 9.93 | 19.46 | 3.75 | 3.28 | 4.22 | 67.7 |
|  | Hyperkinesia* | 35 | 180.17 | 122.34 | 265.35 | 7.04 | 6.64 | 7.45 | 26.33 |
|  | Loss of consciousness* | 35 | 2.9 | 2.08 | 4.05 | 1.53 | 1.05 | 2 | 22 |
|  | Balance disorder* | 34 | 4.21 | 3 | 5.91 | 2.06 | 1.58 | 2.54 | 42 |
|  | Hospitalisation* | 34 | 1.51 | 1.08 | 2.11 | 0.59 | 0.11 | 1.07 | 83.5 |
|  | Infusion site infection* | 34 | 104.78 | 72.35 | 151.76 | 6.43 | 5.99 | 6.87 | 48.33 |
|  | Infusion site swelling* | 31 | 16.65 | 11.63 | 23.83 | 4.01 | 3.51 | 4.51 | 19 |
|  | Cellulitis* | 29 | 5.98 | 4.14 | 8.63 | 2.56 | 2.04 | 3.08 | 81 |
|  | Infusion site cellulitis* | 28 | 537.25 | 314.87 | 916.68 | 8.01 | 7.64 | 8.38 | 3.5 |
|  | Mental disorder* | 28 | 6.1 | 4.2 | 8.87 | 2.59 | 2.06 | 3.12 | 80.29 |
|  | Suicidal ideation* | 28 | 3.26 | 2.25 | 4.73 | 1.7 | 1.16 | 2.23 | 30.33 |
|  | Catheter site pain* | 27 | 77.85 | 51.86 | 116.85 | 6.07 | 5.57 | 6.58 | 12.33 |
|  | Skin infection* | 27 | 17.46 | 11.89 | 25.65 | 4.07 | 3.54 | 4.61 | 54 |
| Disease-expected* | General physical health deterioration* | 111 | 6.14 | 5.08 | 7.41 | 2.59 | 2.32 | 2.85 | 46.05 |
|  | Mobility decreased* | 99 | 10.72 | 8.78 | 13.1 | 3.38 | 3.1 | 3.66 | 52.6 |
|  | Parkinson's disease* | 73 | 29.32 | 23.13 | 37.17 | 4.78 | 4.46 | 5.1 | 30.86 |
|  | Tremor* | 69 | 4.72 | 3.72 | 5.99 | 2.22 | 1.88 | 2.55 | 19.25 |
|  | Freezing phenomenon* | 67 | 113.12 | 86.7 | 147.6 | 6.52 | 6.21 | 6.83 | 46.25 |
|  | Musculoskeletal stiffness* | 62 | 6.11 | 4.75 | 7.85 | 2.59 | 2.23 | 2.94 | 30 |
|  | Gait disturbance* | 59 | 3.09 | 2.39 | 4 | 1.62 | 1.25 | 1.98 | 45.11 |
|  | Asthenia* | 57 | 1.37 | 1.06 | 1.78 | 0.45 | 0.08 | 0.83 | 45.71 |
|  | Movement disorder* | 54 | 17.8 | 13.56 | 23.37 | 4.1 | 3.72 | 4.47 | 58 |
|  | Hypokinesia* | 39 | 34.67 | 25.04 | 47.99 | 5.01 | 4.58 | 5.45 | 52 |
|  | Bradykinesia* | 34 | 30.47 | 21.54 | 43.11 | 4.84 | 4.37 | 5.31 | 10 |
|  | Cognitive disorder* | 34 | 6.62 | 4.72 | 9.29 | 2.71 | 2.22 | 3.19 | 34 |
|  | Dysphagia* | 33 | 3.28 | 2.33 | 4.62 | 1.7 | 1.21 | 2.19 | 56.2 |
|  | Loss of personal independence in daily activities* | 32 | 3.31 | 2.34 | 4.7 | 1.72 | 1.22 | 2.21 | 9.67 |
|  | Muscle rigidity* | 31 | 26.04 | 18.14 | 37.39 | 4.63 | 4.13 | 5.12 | 42.33 |
|  | Gait inability* | 27 | 3.91 | 2.68 | 5.71 | 1.95 | 1.41 | 2.5 | 36.5 |
| Without signal–related | Drug ineffective | 46 | 0.34 | 0.26 | 0.46 | -1.53 | -1.94 | -1.11 | 39.6 |
|  | Fatigue | 44 | 0.46 | 0.34 | 0.61 | -1.12 | -1.55 | -0.7 | 54.4 |
|  | Pyrexia | 40 | 0.98 | 0.72 | 1.34 | -0.02 | -0.47 | 0.42 | 14.75 |
|  | Dizziness | 39 | 0.77 | 0.56 | 1.06 | -0.37 | -0.82 | 0.08 | 20.33 |
|  | Weight decreased | 38 | 1.17 | 0.85 | 1.61 | 0.23 | -0.23 | 0.68 | 73.4 |
|  | Pain | 35 | 0.57 | 0.41 | 0.79 | -0.81 | -1.28 | -0.33 | 29.5 |
|  | Dyspnoea | 33 | 0.52 | 0.37 | 0.74 | -0.93 | -1.42 | -0.44 | 3.5 |
|  | Nausea | 29 | 0.36 | 0.25 | 0.51 | -1.48 | -2 | -0.95 | 25.86 |

PT, Preferred Term; CI, confidence interval; ROR, Reporting Odds Ratio; IC, Information Component.
