# Supplementary material for: Post-marketing safety concerns with foscarbidopa/foslevodopa: A pharmacovigilance study with disproportionality analysis based on FAERS
Source: Medicine (Baltimore). 2026 May 15;105(20):e48874. doi: 10.1097/MD.0000000000048874 (PMC13183012; doi:10.1097/MD.0000000000048874)
Supplement: Supplementary file 3 [file medi-105-e48874-s003.docx]

Table S3a Reporting Odds Ratios and Information Components for top 60 foscarbidopa/foslevodopa-related Adverse Events in Males.

| PT | No. | ROR | Lower  95%CI | Upper 95%CI | IC | Lower  95%CI | Upper 95%CI |
| --- | --- | --- | --- | --- | --- | --- | --- |
| On and off phenomenon* | 125 | 125.87 | 101.56 | 156.00 | 6.38 | 6.18 | 6.59 |
| Fall* | 106 | 4.79 | 3.94 | 5.82 | 2.21 | 1.94 | 2.48 |
| Hallucination* | 85 | 10.75 | 8.63 | 13.38 | 3.35 | 3.05 | 3.65 |
| General physical health deterioration* | 70 | 8.22 | 6.47 | 10.45 | 2.98 | 2.65 | 3.31 |
| Dyskinesia* | 66 | 27.20 | 21.07 | 35.10 | 4.61 | 4.28 | 4.94 |
| Mobility decreased* | 55 | 14.22 | 10.82 | 18.68 | 3.74 | 3.37 | 4.11 |
| Malaise* | 50 | 2.49 | 1.89 | 3.30 | 1.30 | 0.90 | 1.70 |
| Pneumonia* | 50 | 1.90 | 1.44 | 2.51 | 0.91 | 0.52 | 1.31 |
| Infusion site pain* | 48 | 39.71 | 29.27 | 53.87 | 5.10 | 4.72 | 5.48 |
| Parkinson's disease* | 44 | 18.83 | 13.84 | 25.60 | 4.13 | 3.72 | 4.54 |
| Musculoskeletal stiffness* | 42 | 9.93 | 7.28 | 13.53 | 3.25 | 2.82 | 3.68 |
| Tremor* | 42 | 4.96 | 3.65 | 6.74 | 2.28 | 1.85 | 2.71 |
| Therapeutic product effect incomplete* | 41 | 5.86 | 4.29 | 8.00 | 2.51 | 2.08 | 2.95 |
| Confusional state* | 40 | 4.08 | 2.98 | 5.58 | 2.00 | 1.56 | 2.44 |
| Device issue* | 40 | 4.88 | 3.57 | 6.69 | 2.26 | 1.81 | 2.70 |
| Freezing phenomenon* | 40 | 65.45 | 46.23 | 92.65 | 5.70 | 5.31 | 6.10 |
| Infusion site reaction* | 40 | 159.66 | 107.68 | 236.74 | 6.63 | 6.28 | 6.98 |
| Gait disturbance* | 39 | 3.70 | 2.70 | 5.09 | 1.86 | 1.42 | 2.31 |
| Infusion site erythema* | 39 | 44.46 | 31.62 | 62.51 | 5.24 | 4.83 | 5.66 |
| Infusion site induration* | 36 | 364.43 | 219.86 | 604.07 | 7.25 | 6.95 | 7.55 |
| Movement disorder* | 33 | 19.11 | 13.40 | 27.25 | 4.15 | 3.68 | 4.62 |
| Aggression* | 31 | 7.50 | 5.24 | 10.73 | 2.86 | 2.36 | 3.36 |
| Asthenia | 31 | 1.31 | 0.92 | 1.87 | 0.39 | -0.12 | 0.89 |
| Infusion site abscess* | 31 | 679.13 | 348.54 | 1323.29 | 7.56 | 7.30 | 7.83 |
| Urinary tract infection* | 29 | 3.41 | 2.36 | 4.93 | 1.75 | 1.23 | 2.27 |
| Anxiety* | 28 | 2.28 | 1.57 | 3.31 | 1.18 | 0.65 | 1.71 |
| Infusion site inflammation* | 28 | 350.27 | 198.76 | 617.27 | 7.23 | 6.88 | 7.57 |
| Fatigue | 27 | 0.58 | 0.40 | 0.85 | -0.77 | -1.32 | -0.23 |
| Feeling abnormal* | 27 | 3.23 | 2.21 | 4.73 | 1.67 | 1.13 | 2.21 |
| Infection* | 26 | 2.47 | 1.67 | 3.63 | 1.29 | 0.74 | 1.84 |
| Drug ineffective | 24 | 0.31 | 0.21 | 0.46 | -1.68 | -2.25 | -1.10 |
| Somnolence* | 24 | 1.98 | 1.33 | 2.97 | 0.98 | 0.40 | 1.55 |
| Hyperkinesia* | 23 | 107.76 | 66.26 | 175.26 | 6.25 | 5.76 | 6.75 |
| Hypokinesia* | 23 | 39.96 | 25.74 | 62.03 | 5.11 | 4.57 | 5.66 |
| Dizziness | 22 | 0.91 | 0.60 | 1.38 | -0.14 | -0.74 | 0.46 |
| Balance disorder* | 21 | 4.39 | 2.85 | 6.77 | 2.11 | 1.50 | 2.72 |
| Bradykinesia* | 21 | 20.78 | 13.31 | 32.44 | 4.27 | 3.67 | 4.86 |
| Hallucination, visual* | 21 | 9.76 | 6.31 | 15.10 | 3.23 | 2.63 | 3.84 |
| Infusion site nodule* | 21 | 262.26 | 143.13 | 480.57 | 7.03 | 6.60 | 7.47 |
| Pyrexia | 21 | 0.81 | 0.53 | 1.24 | -0.30 | -0.92 | 0.31 |
| Weight decreased | 21 | 1.05 | 0.68 | 1.62 | 0.07 | -0.54 | 0.69 |
| Cognitive disorder* | 20 | 5.52 | 3.54 | 8.61 | 2.43 | 1.81 | 3.06 |
| Dysphagia* | 20 | 3.20 | 2.05 | 4.97 | 1.66 | 1.03 | 2.29 |
| Hospitalisation | 20 | 1.26 | 0.81 | 1.96 | 0.33 | -0.29 | 0.96 |
| Infusion site infection* | 20 | 138.00 | 80.23 | 237.35 | 6.50 | 5.99 | 7.01 |
| Loss of consciousness* | 20 | 2.54 | 1.63 | 3.95 | 1.33 | 0.70 | 1.96 |
| Pain | 20 | 0.74 | 0.48 | 1.15 | -0.43 | -1.06 | 0.20 |
| Akinesia* | 19 | 191.56 | 105.94 | 346.38 | 6.79 | 6.30 | 7.28 |
| Unevaluable event* | 18 | 5.63 | 3.53 | 8.99 | 2.46 | 1.81 | 3.12 |
| Infusion site cellulitis* | 17 | 556.79 | 240.16 | 1290.86 | 7.48 | 7.09 | 7.86 |
| Sepsis* | 17 | 1.98 | 1.23 | 3.20 | 0.98 | 0.30 | 1.66 |
| Agitation* | 16 | 3.65 | 2.22 | 5.98 | 1.85 | 1.15 | 2.55 |
| Catheter site pain* | 16 | 77.61 | 44.39 | 135.70 | 5.90 | 5.28 | 6.52 |
| Cellulitis* | 16 | 5.38 | 3.28 | 8.84 | 2.40 | 1.70 | 3.10 |
| Loss of personal independence in daily activities* | 16 | 3.07 | 1.88 | 5.04 | 1.60 | 0.90 | 2.31 |
| Skin infection* | 16 | 20.34 | 12.22 | 33.86 | 4.24 | 3.56 | 4.92 |
| Infusion site swelling* | 15 | 19.35 | 11.44 | 32.72 | 4.17 | 3.47 | 4.88 |
| Muscle rigidity* | 15 | 13.27 | 7.89 | 22.31 | 3.66 | 2.95 | 4.37 |
| Psychotic disorder* | 15 | 6.60 | 3.95 | 11.03 | 2.69 | 1.97 | 3.41 |
| Speech disorder* | 15 | 4.89 | 2.93 | 8.15 | 2.26 | 1.54 | 2.99 |

Abbreviations: * Signal detected; PT, Preferred Terms; CI, confidence interval; ROR, reporting odds ratio; IC, information component.
