# Supplementary material for: Post-marketing safety concerns with foscarbidopa/foslevodopa: A pharmacovigilance study with disproportionality analysis based on FAERS
Source: Medicine (Baltimore). 2026 May 15;105(20):e48874. doi: 10.1097/MD.0000000000048874 (PMC13183012; doi:10.1097/MD.0000000000048874)
Supplement: Supplementary file 4 [file medi-105-e48874-s004.docx]

Table S3b Reporting Odds Ratios and Information Components for top 60 foscarbidopa/foslevodopa-related Adverse Events in Females.

| PT | No. | ROR | Lower  95%CI | Upper 95%CI | IC | Lower  95%CI | Upper 95%CI |
| --- | --- | --- | --- | --- | --- | --- | --- |
| On and off phenomenon* | 95 | 379.33 | 294.40 | 488.75 | 7.89 | 7.66 | 8.11 |
| Fall* | 87 | 5.83 | 4.70 | 7.22 | 2.50 | 2.20 | 2.79 |
| Hallucination* | 67 | 23.89 | 18.67 | 30.56 | 4.50 | 4.16 | 4.83 |
| Dyskinesia* | 65 | 51.00 | 39.53 | 65.79 | 5.54 | 5.20 | 5.87 |
| Infusion site erythema* | 45 | 35.90 | 26.54 | 48.56 | 5.07 | 4.66 | 5.48 |
| Mobility decreased* | 44 | 10.24 | 7.58 | 13.82 | 3.32 | 2.90 | 3.74 |
| General physical health deterioration* | 41 | 7.13 | 5.23 | 9.72 | 2.80 | 2.37 | 3.24 |
| Infusion site pain* | 35 | 22.01 | 15.69 | 30.88 | 4.40 | 3.93 | 4.87 |
| Infusion site reaction* | 35 | 75.02 | 52.80 | 106.60 | 6.06 | 5.61 | 6.51 |
| Malaise* | 33 | 1.70 | 1.21 | 2.40 | 0.76 | 0.27 | 1.25 |
| Device issue* | 30 | 6.10 | 4.25 | 8.75 | 2.58 | 2.07 | 3.10 |
| Infusion site induration* | 30 | 241.34 | 158.72 | 366.99 | 7.46 | 7.02 | 7.90 |
| Urinary tract infection* | 30 | 2.70 | 1.88 | 3.87 | 1.42 | 0.91 | 1.93 |
| Parkinson's disease* | 29 | 38.94 | 26.73 | 56.72 | 5.19 | 4.68 | 5.70 |
| Confusional state* | 28 | 4.37 | 3.00 | 6.34 | 2.11 | 1.58 | 2.64 |
| Freezing phenomenon* | 27 | 151.34 | 99.50 | 230.19 | 6.94 | 6.45 | 7.42 |
| Tremor* | 27 | 4.50 | 3.07 | 6.58 | 2.15 | 1.61 | 2.69 |
| Asthenia* | 25 | 1.59 | 1.07 | 2.35 | 0.66 | 0.10 | 1.22 |
| Infusion site abscess* | 25 | 980.30 | 528.82 | 1817.23 | 8.62 | 8.27 | 8.98 |
| Infusion site nodule* | 24 | 235.19 | 147.47 | 375.08 | 7.43 | 6.94 | 7.93 |
| Pneumonia | 23 | 1.32 | 0.87 | 1.98 | 0.39 | -0.19 | 0.98 |
| Anxiety* | 22 | 1.83 | 1.20 | 2.78 | 0.86 | 0.26 | 1.46 |
| Therapeutic product effect incomplete* | 22 | 4.71 | 3.09 | 7.17 | 2.22 | 1.62 | 2.82 |
| Gait disturbance* | 20 | 2.47 | 1.59 | 3.84 | 1.30 | 0.67 | 1.92 |
| Movement disorder* | 20 | 15.34 | 9.84 | 23.94 | 3.90 | 3.28 | 4.52 |
| Dyspnoea | 19 | 0.80 | 0.51 | 1.26 | -0.32 | -0.96 | 0.33 |
| Musculoskeletal stiffness* | 19 | 3.91 | 2.49 | 6.15 | 1.95 | 1.31 | 2.60 |
| Akinesia* | 18 | 266.01 | 153.81 | 460.05 | 7.56 | 7.00 | 8.12 |
| Drug ineffective | 18 | 0.38 | 0.24 | 0.61 | -1.37 | -2.03 | -0.70 |
| Pyrexia | 18 | 1.17 | 0.73 | 1.86 | 0.22 | -0.44 | 0.89 |
| Suicidal ideation* | 18 | 5.53 | 3.47 | 8.80 | 2.45 | 1.79 | 3.11 |
| Fatigue | 17 | 0.44 | 0.27 | 0.70 | -1.18 | -1.87 | -0.50 |
| Somnolence* | 17 | 2.21 | 1.37 | 3.56 | 1.14 | 0.45 | 1.82 |
| Weight decreased | 17 | 1.34 | 0.83 | 2.17 | 0.42 | -0.26 | 1.11 |
| Hypokinesia* | 16 | 34.08 | 20.59 | 56.39 | 5.01 | 4.33 | 5.70 |
| Infection* | 16 | 1.86 | 1.14 | 3.04 | 0.89 | 0.19 | 1.59 |
| Infusion site inflammation* | 16 | 212.66 | 120.97 | 373.84 | 7.33 | 6.71 | 7.94 |
| Muscle rigidity* | 16 | 42.19 | 25.42 | 70.02 | 5.30 | 4.62 | 5.99 |
| Nausea | 16 | 0.42 | 0.26 | 0.69 | -1.24 | -1.94 | -0.53 |
| Delusion* | 15 | 29.39 | 17.50 | 49.36 | 4.81 | 4.10 | 5.52 |
| Dizziness | 15 | 0.69 | 0.42 | 1.15 | -0.53 | -1.25 | 0.20 |
| Infusion site swelling* | 15 | 14.91 | 8.93 | 24.91 | 3.86 | 3.14 | 4.58 |
| Loss of consciousness* | 15 | 3.27 | 1.97 | 5.44 | 1.70 | 0.97 | 2.43 |
| Loss of personal independence in daily activities* | 15 | 3.78 | 2.27 | 6.29 | 1.91 | 1.18 | 2.63 |
| Pain | 15 | 0.52 | 0.31 | 0.86 | -0.94 | -1.67 | -0.21 |
| Gait inability* | 14 | 4.18 | 2.47 | 7.07 | 2.05 | 1.30 | 2.80 |
| Balance disorder* | 13 | 4.05 | 2.35 | 7.00 | 2.01 | 1.23 | 2.79 |
| Dysphagia* | 13 | 3.47 | 2.01 | 5.98 | 1.78 | 1.00 | 2.56 |
| Hallucination, visual* | 13 | 15.72 | 9.06 | 27.27 | 3.94 | 3.16 | 4.71 |
| Hospitalisation | 13 | 1.69 | 0.98 | 2.92 | 0.75 | -0.03 | 1.54 |
| Infusion site infection* | 13 | 73.14 | 41.20 | 129.83 | 6.04 | 5.30 | 6.78 |
| Mental disorder* | 13 | 7.45 | 4.31 | 12.88 | 2.88 | 2.10 | 3.66 |
| Back pain | 12 | 1.17 | 0.66 | 2.07 | 0.23 | -0.59 | 1.04 |
| Bradykinesia* | 12 | 33.18 | 18.56 | 59.31 | 4.98 | 4.18 | 5.77 |
| Cognitive disorder* | 12 | 6.50 | 3.68 | 11.50 | 2.68 | 1.87 | 3.49 |
| Delirium* | 12 | 7.34 | 4.15 | 12.97 | 2.86 | 2.05 | 3.67 |
| Disorientation* | 12 | 11.10 | 6.27 | 19.67 | 3.45 | 2.64 | 4.25 |
| Hyperkinesia* | 12 | 204.21 | 106.82 | 390.39 | 7.28 | 6.57 | 8.00 |
| Infusion site discharge* | 12 | 20.63 | 11.60 | 36.68 | 4.32 | 3.52 | 5.12 |
| Injection site erythema* | 12 | 1.94 | 1.10 | 3.42 | 0.95 | 0.14 | 1.76 |

Abbreviations: * Signal detected; PT, Preferred Terms; CI, confidence interval; ROR, reporting odds ratio; IC, information component.
